# 固相萃取净化-超高效液相色谱-高分辨质谱法测定尿液中百草枯和敌草快残留

**DOI:** 10.3724/SP.J.1123.2022.02012

**Published:** 2022-12-08

**Authors:** Shengdong PAN, Li WANG, Qiaoli QIU, Qian HE

**Affiliations:** 1.宁波市疾病预防控制中心, 浙江省微量有毒化学物健康风险评估技术研究重点实验室, 浙江 宁波 315010; 1. Key Laboratory of Health Risk Appraisal for Trace Toxic Chemicals of Zhejiang Province, Ningbo Municipal Center for Disease Control and Prevention, Ningbo 315010, China; 2.中国疾病预防控制中心, 职业卫生与中毒控制所, 北京 100050; 2. National Institute of Occupational Health and Poison Control, Chinese Municipal Center for Disease Control and Prevention, Beijing 100050, China

**Keywords:** 超高效液相色谱-高分辨质谱, 弱阳离子交换固相萃取, 百草枯, 敌草快, 尿液样品, 基质效应, ultra performance liquid chromatography-high resolution mass spectrometry (UPLC-HRMS), weak cation exchange (WCX) solid-phase extraction (SPE), paraquat (PQ), diquat (DQ), urine samples, matrix effect

## Abstract

尿液样品中百草枯(PQ)和敌草快(DQ)的检测是理化检验工作的难点。PQ和DQ具有分子极性大和水溶性好等特点,常规反相色谱柱难以保留;现有文献方法多采用亲水相互作用色谱法(HILIC)进行保留,但文献方法需采用高浓度缓冲盐作为流动相,增加了质谱仪的污染。基于上述问题,研究建立了弱阳离子交换(WCX)固相萃取净化-超高效液相色谱-高分辨质谱法(UPLC-HRMS)快速准确测定尿液样品中PQ和DQ残留的检测方法。尿液样品经混合磷酸盐缓冲液(pH=6.86)稀释和WCX固相萃取净化后,在Syncronis HILIC色谱柱(100 mm×2.1 mm, 1.7 μm)上进行梯度洗脱分离,采用正离子电喷雾离子化模式(ESI^+^)和一级全扫描-数据依赖二级质谱扫描模式(Full mass-ddMS^2^)进行定量分析。研究通过对色谱条件的不断优化,将HILIC模式下流动相中甲酸铵缓冲盐的浓度降低至10 mmol/L,并系统优化了样品前处理过程中影响PQ和DQ准确性的因素。在最优条件下,PQ和DQ线性关系良好(*r*^2^>0.998),在4个加标水平下(1.0、20.0、100.0和200.0 μg/L), PQ和DQ的平均加标回收率分别为85.8%~101%和80.3%~86.9%,精密度(RSD)分别为0.8%~5.1%和0.9%~4.2%。方法的检出限(*S/N*≥3)和定量限(*S/N*≥10)分别为0.2 μg/L和0.6 μg/L。将建立的方法用于中毒病人临床治疗过程尿液中DQ含量的跟踪监测。该方法具有快速、简便、灵敏和准确等优点,适用于临床中毒病例尿液样品中PQ和DQ的检测。

## Abstract (en)

Determining the presence of paraquat (PQ) and diquat (DQ) in urine samples through physical and chemical testing is challenging. As PQ and DQ have characteristics such as high molecular polarity and good water solubility, they are difficult to be retained by conventional reversed-phase columns. Most of the methods in the literature use hydrophilic interaction chromatography (HILIC) for the retention of PQ and DQ, but they often require high concentrations of buffer salts as the mobile phase, which increase the contamination of the mass spectrometer. In view of the above problems, a rapid and accurate analysis method was developed for the determination of PQ and DQ residuals in urine samples based on weak cation exchange (WCX) solid-phase extraction (SPE) and ultra performance liquid chromatography-high resolution mass spectrometry (UPLC-HRMS) in this study. Urine samples were first diluted with phosphate buffer (pH=6.86) and pretreated using the WCX SPE method. Chromatographic separation was performed on a Syncronis HILIC column (100 mm×2.1 mm, 1.7 μm). An electrospray ion source in the positive (ESI^+^) mode and full mass-data dependent MS^2^ (full mass-ddMS^2^) mode was used for quantification by matrix-matched external standard method. In this study, the concentration of ammonium formate in the mobile phase in the HILIC mode was effectively reduced to 10 mmol/L by the continuous optimization of the chromatographic conditions. MS optimization results indicated that the molecular ion (M^+·^) of PQ and DQ had the strongest response. In addition, sample pretreatment conditions were also optimized. The obtained results indicated that the hydrophobic polytetrafluoroethylene (PTFE) filter membrane, acetonitrile-water (1∶1, v/v) as a fixing solution, and polypropylene vials were suitable for PQ and DQ analysis. Under the optimal conditions, the linearity of PQ and DQ was good with correlation coefficients (*r*^2^) greater than 0.998. The limits of detection (LODs, *S/N*≥3) and limits of quantification (LOQs, *S/N*≥10) were 0.2 μg/L and 0.6 μg/L, respectively. Mean spiked recoveries of PQ and DQ at the four spiked levels (1.0, 20.0, 100.0, and 200.0 μg/L) were in the range of 85.8%-101% and 80.3%-86.9%, with the RSDs of 0.8%-5.1% and 0.9%-4.2%. The established method was employed for the analysis and confirmation of PQ and DQ for clinical poisoning cases. In one case, a 23-year-old male who had taken approximately 20 mL of pesticide orally was confirmed as DQ poisoning by the developed method. DQ concentration monitoring of the urine samples was conducted for this case during the clinical treatment process. The patient was successfully discharged from the hospital after five times of blood perfusion and other treatments until the DQ concentration was low in the urine samples. In conclusion, the method developed in this study based on WCX SPE-UPLC-HRMS can be used for the confirmation of poisoning cases and concentration monitoring during clinical treatment, providing strong technical support for clinical precision treatment. The method is rapid, simple, sensitive, and accurate, and it is suitable for the detection of PQ and DQ in urine samples.

百草枯(paraquat, PQ)和敌草快(diquat, DQ)同属快速灭生性除草剂,对人体毒性极大,临床致死率高,毒性可累及全身多个脏器,可导致多器官功能障碍,至今尚无特效解毒药^[1]^。PQ的主要作用靶点为肺部,可使中毒患者肺纤维化,致使患者因缺氧而窒息死亡。目前,我国虽已禁止PQ的生产与使用,但由于管理难度太大,仍有不法商贩在经营销售。另一方面,随着PQ的全面禁用,DQ的市场份额逐年增长,尽管DQ的毒性和致死率不及PQ,但高剂量DQ中毒死亡率仍居高不下。近年来,因自杀、误服和投毒而引起的PQ和DQ急性中毒事件量呈逐年上升趋势,已成为我国最为主要的中毒致死农药之一^[2]^。因此,开展中毒生物样本中PQ和DQ的快速确证与定量分析具有重要的临床价值和现实意义。

尽管血浆中PQ和DQ的浓度水平在预后方面具有更高的预测价值,但血浆样本中浓度往往较低,检测过程中基质效应会更加显著,不利于临床中毒确证分析。与此同时,PQ和DQ在肾小管中不被重吸收,主要以原形从肾脏排出^[3,4]^,因此中毒病人尿液中PQ和DQ的浓度比较高,开展尿液样品中毒物的检测有利于快速准确评判毒物种类与毒物在体内的排泄情况^[5]^。迄今,中毒生物样本中PQ和DQ的检测方法主要包括毛细管电泳法(CE)^[6]^、液相色谱-紫外检测法(LC-UV)^[7]^、气相色谱-质谱法(GC-MS)^[8]^和液相色谱-质谱法(LC-MS)^[9⇓-11]^。其中,CE仪器在实验室的普及率不高,且该方法存在灵敏度低的缺点;LC-UV由于仪器自身的局限性,常常会提供假阳性结果;GC-MS需要衍生化后方能准确测定,操作过程复杂、影响因素较多。张秀尧等^[12]^开发了离子色谱-质谱测定血浆和尿液中PQ和DQ的分析方法,得到了令人满意的效果,但限于离子色谱-质谱联用仪的低普及率,难以在普通理化实验室得到有效开展。因此,开发普适性高和操作简便的检测方法显得尤为重要。因LC-MS具有高灵敏度、高准确性等优点,基于LC-MS检测中毒生物样本中有毒有害物质的方法越来越受到关注。尤其近年来随着液相色谱-高分辨质谱技术(LC-HRMS)的高速发展和仪器的普及,利用精确质量数定性与定量所体现的优势为中毒生物样品中有毒有害物质的快速测定提供了有效的解决方案^[9]^。尤其对于相对分子质量较低和极性较强的PQ和DQ的检测,LC-HRMS能弥补液相色谱-串联质谱(LC-MS/MS)定性能力差的不足。此外,由于PQ和DQ强极性的特点,传统的反相色谱柱难以有效保留,需使用七氟丁酸等离子对试剂提高PQ和DQ在反相色谱柱上的保留能力^[13]^,但离子对试剂易对质谱仪产生污染,且会抑制目标分析物的质谱响应。近年来,许多研究者采用亲水相互作用色谱柱(HILIC)实现了PQ和DQ的有效保留^[14,15]^,但大多研究都在流动相中加入高浓度的甲酸铵缓冲盐(可高至250 mmol/L),同样给质谱仪增加了额外的负担。与此同时,在LC-MS检测过程中基质干扰效应是不可忽视的影响因素,处理不当会严重影响检测结果的准确性^[16]^,需要选择合适的样品前处理技术对中毒生物样品进行有效净化,进而达到准确测定的目的。目前,对于生物样品中PQ和DQ的前处理技术主要包括液液萃取法(LLE)^[17]^、固相微萃取法(SPME)^[18]^和固相萃取法(SPE)^[11,19]^。其中,LLE虽然操作简便快速,但该法难以有效消除基质效应;SPME需要额外增加顶空进样系统,且该方法吸附容量有限;商品化的SPE小柱种类多样,如C_18_、HLB、MCX、MAX等,根据待测化合物的结构特点,选择合适的SPE小柱,可满足大多数化合物的净化需求^[19]^。

本研究选择临床致死率高、分子极性大、检测难度高的PQ和DQ为研究对象,通过不断优化色谱条件,在HILIC模式下,有效降低了流动相中缓冲盐的浓度(10 mmol/L),并改善了PQ和DQ的色谱峰形,克服了PQ和DQ传统LC-MS分析方法使用高浓度缓冲盐的不足,为PQ和DQ提供了全新的检测方案。此外,本研究通过精细的实验设计,系统考察了PQ和DQ样品预处理过程中影响检测准确度的主要因素,如滤膜的选择、进样瓶材质的影响、定容液组成和上样液pH值的影响等,为检测人员提供了系统详实的实验结果与科学合理的理论阐释。

## 1 实验部分

### 1.1 仪器、试剂与材料

Waters UPLC I Class型超高效液相色谱仪(美国Waters公司); Q-Exactive Orbitrap型高分辨质谱仪(美国Thermo Fisher公司)和Trace Finder 3.3数据处理系统;低温高速离心机(德国Sigma公司); 12位固相萃取装置(美国Agilent公司);超低温冰箱(浙江捷盛低温设备有限公司)。

乙腈(HPLC级)和甲酸(LC-MS级)均购自美国Thermo Fisher公司;甲酸铵(HPLC级)购自上海安谱有限公司;百草枯二氯化物和敌草快二溴化物标准品均购自德国Dr. Ehrenstorfer公司;邻苯二甲酸氢钾缓冲液(pH=4.00)、混合磷酸盐缓冲液(pH=6.86)和硼酸缓冲液(pH=9.18)均购自上海爱建试剂厂有限公司,临用前采用250 mL蒸馏水溶解并定容即可;实验用水经Milli-Q超纯水仪(法国Millipore公司)制备; 混合型强阳离子交换(MCX)固相萃取柱(60 mg/3 mL)和弱阳离子交换(WCX)固相萃取柱(60 mg/3 mL)均购自美国Waters公司。

中毒尿样和血样由宁波市北仑区人民医院提供,该中毒病例为23岁男性,于2019年9月5日12时30分口服敌草快水剂约20 mL,当天下午18∶00入院,治疗前采集约10 mL尿液样本于塑料尿杯中,分别于2019年9月5日下午20∶22~9月8日10∶30进行5次血液灌流治疗,其中第1、2、5次血液灌流治疗后以及第5次血液灌流治疗后24 h分别采集尿样。空白尿样由志愿者提供。本研究进行的所有程序都符合伦理标准,并经宁波市疾病预防控制中心伦理审查委员会批准,批准通知书编号为201804和202006。

### 1.2 实验方法

#### 1.2.1 样品前处理

将采集的尿液样本采用8000 r/min离心5 min除去杂质,收集上清液于10 mL塑料离心管中,置于-80 ℃超低温冰箱保存。

准确移取解冻的尿样1.0 mL于15 mL聚丙烯离心管中,加入9 mL pH=6.86混合磷酸盐缓冲液,涡旋混匀30 s。然后将稀释后的样品溶液加入经3 mL甲醇和3 mL纯水活化与平衡过的WCX固相萃取小柱净化,依次用5 mL水和5 mL甲醇淋洗,弃去所有淋洗液,之后用5 mL甲酸-乙腈(2∶98, v/v)洗脱,收集洗脱液,于40 ℃氮吹至干,加入1.0 mL乙腈-水(1∶1, v/v)溶解残渣,经0.22 μm疏水性聚四氟乙烯(PTFE)滤膜过滤后,UPLC-HRMS测定。

#### 1.2.2 色谱-质谱条件

色谱柱为Syncronis HILIC色谱柱(100 mm×2.1 mm, 1.7 μm);流动相:A相,0.25%(v/v)甲酸水溶液(10 mmol/L甲酸铵); B相,乙腈。梯度洗脱:0~2.00 min, 80%B; 2.00~2.50 min, 80%B~20%B; 2.50~5.00 min, 20%B; 5.00~5.01 min, 20%B~80%B; 5.01~8.00 min, 80%B。流速0.3 mL/min;进样量5 μL;柱温40 ℃。

离子源:电喷雾正离子模式(ESI^+^);离子传输管温度:320 ℃;定量检测方式:一级全扫描-数据依赖二级质谱扫描模式(Full mass-ddMS^2^);一级质谱全扫描范围:*m/z* 100~300;分辨率:全扫描(Full Mass)70000,二级质谱扫描(MS/MS)17500;隔离窗口(Isolation window): *m/z* 2.0;电喷雾电压:3800 V;鞘气压力:275.8 kPa;辅助气速率:180 L/h;反吹气压力:13.8 kPa;辅助气加热温度:200 ℃;射频棱镜设置值(S-lens RF level): 50%。其他质谱参数列于表1。

**表1:** PQ和DQ的分子式、电离形式和定量离子

Compound	Molecular formula	Ionized form	Quantitative ions (m/z)
Paraquat (PQ)	C_12_H_14_N_2_	M^+·^	186.11515
Diquat (DQ)	C_12_H_12_N_2_	M^+·^	184.09950

#### 1.2.3 标准溶液的配制

分别准确称取10.0 mg PQ和DQ标准物质,用乙腈-水(1∶1, v/v)配制成1.0 mg/mL单标准溶液。分别准确吸取适量PQ和DQ单标准溶液,用乙腈-水(1∶1, v/v)配制成10 mg/L混合标准溶液。

吸取适量10 mg/L混合标准溶液,采用经处理的空白尿样净化液配制PQ和DQ质量浓度分别为1.0、5.0、10.0、50.0、100.0和200.0 μg/L的基质标准溶液。

## 2 结果与讨论

### 2.1 一级质谱图解析

由于特殊的化学结构,PQ和DQ的质谱行为较为复杂。一方面,PQ和DQ都为双电荷化合物,其一级质谱图上存在明显分子离子M^2+^的质谱信号,即PQ和DQ的分子离子 $M_{1}^{2+}$ 和 $M_{2}^{2+}$ 的*m/z*分别为93.05716和92.04931(见图1a),与理论精确质量数的偏差分别为1.5×10^-6^和1.8×10^-6^;另一方面,在一级质谱图上也存在较强的分子离子M^+·^的质谱信号,其精确质量数分别为186.11481和184.09921(见图1b),与理论精确质量数的偏差分别为1.8×10^-6^和1.6×10^-6^;此外,PQ和DQ易发生源内的*α*-裂解形成准分子离子[M^2+^-H^+^]^+^信号,其精确质量数分别为185.10732和183.09142(见图1b),与理论精确质量数的偏差分别为2.3×10^-6^和1.5×10^-6^。现有大多文献^[20,21]^选择PQ和DQ的[M^2+^-H^+^]^+^质谱信号作为母离子进行定量分析。但从本研究Q-Exactive高分辨质谱一级质谱图来看,相比M^2+^和[M^2+^-H^+^]^+^, PQ和DQ的分子离子M^+·^的质谱信号最强,且质量偏差均小于3.0×10^-6^。因此,后续研究均采用分子离子M^+·^作为一级定量离子,具有质量精度高、定性能力强等优点。

**图1:**
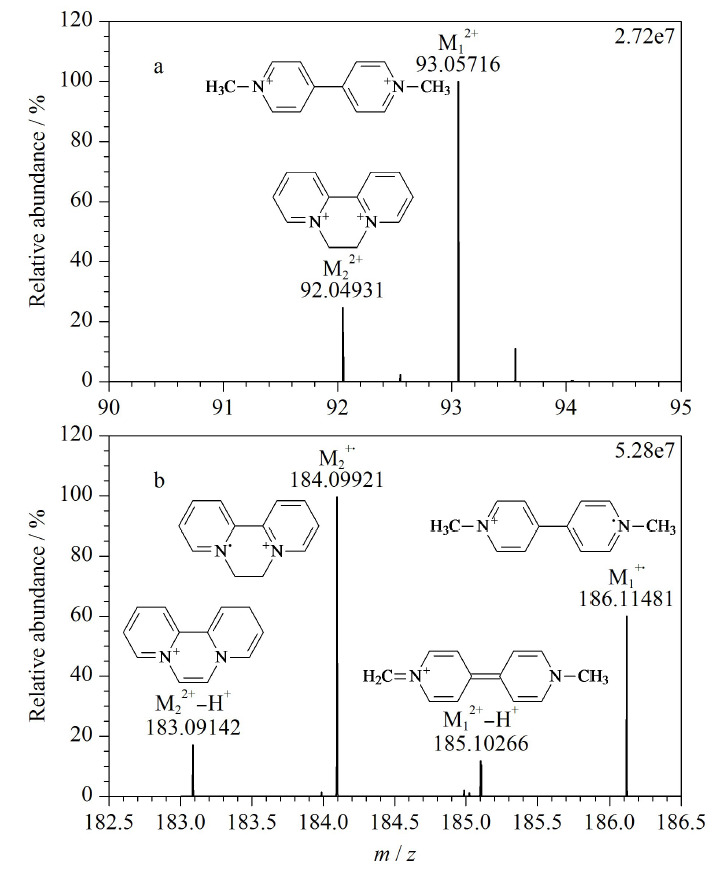
PQ和DQ的一级质谱图

### 2.2 液相色谱条件优化

PQ和DQ均为联吡啶季铵阳离子,特殊的化学结构给色谱分析与检测工作带来了巨大的挑战,如普遍存在色谱峰拖尾、峰形宽和色谱保留差等不足。本课题组前期的研究工作采用SIELC Obelisc R色谱柱在等度洗脱条件下分离分析尿液中PQ与DQ^[9]^。实验结果表明,相比Waters ACQUITY UPLC HILIC色谱柱和Dikma Polyamino HILIC色谱柱,采用SIELC Obelisc R色谱柱时PQ和DQ的色谱峰形更佳。但使用SIELC Obelisc R色谱柱时,水相流动相中缓冲盐甲酸铵的浓度需控制在50 mmol/L以上,对质谱仪不够友好,易造成喷雾针堵塞;且需要严格调节流动相溶液pH值至3.7,配制过程较为繁琐。此外,采用SIELC Obelisc R色谱柱分析PQ和DQ时,两种化合物的峰宽仍在1 min以上。鉴于此,本研究工作进一步对色谱柱和流动相组分进行优化,实验结果表明,采用Syncronis HILIC色谱柱分析时,PQ与DQ的色谱峰形对称,峰宽窄(约为0.3 min)(见图2);且相比前期研究工作需采用高浓度缓冲盐,本研究将水相中甲酸铵浓度降低至10 mmol/L,并控制甲酸体积分数为0.25%。因此,经优化后的流动相组分对UPLC-HRMS仪器更加友好,且流动相配制过程更加简单。

**图2:**
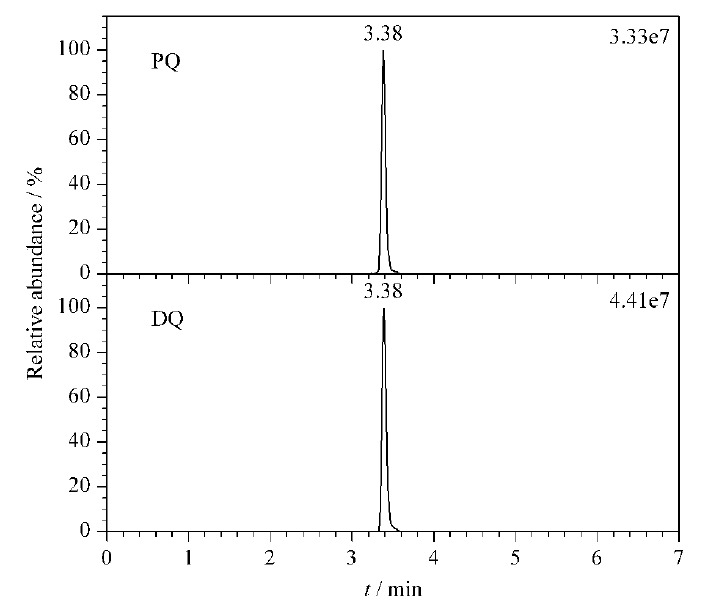
PQ和DQ标准溶液的色谱图

### 2.3 样品前处理条件的优化

#### 2.3.1 滤膜的选择

滤膜在样品前处理过程中起着重要的作用,尽管高速离心能去除大部分固体颗粒,但有时候胶体状的杂质难以用离心过程除去,而滤膜能有效消除这些杂质的影响。然而,在滤膜使用过程中必须要考虑对目标化合物有无吸附作用,即经滤膜处理后样品溶液中目标化合物是否会有损失。

基于此,本研究分别考察了标准溶液不过滤膜以及该标准溶液分别过疏水性PTFE滤膜、亲水性PTFE滤膜和尼龙滤膜4种方式对PQ和DQ峰面积的影响,结果见图3。由图3可知,样品处理过程中滤膜的种类对PQ和DQ峰面积有显著影响,其中经亲水性PTFE滤膜过滤后PQ和DQ几乎已全部被吸附,可能的原因是亲水性PTFE滤膜的原材料中含有聚乙二醇等组分^[22]^,即滤膜表面含有的羟基等活性基团能吸附样品溶液中带正电的PQ和DQ,从而导致PQ和DQ在过滤过程中的损失;标准溶液经尼龙滤膜过滤后,PQ和DQ的峰面积相比未过膜溶液有了显著的增加,可能的原因是尼龙滤膜中存在某种能导致PQ和DQ在LC-MS检测过程中基质增强的成分;相比而言,疏水性PTFE滤膜对标准溶液中PQ和DQ的峰面积没有显著的影响,其峰面积分别为未过滤膜时的96.2%和98.2%。因此,后续实验均采用疏水性PTFE滤膜过滤样品溶液。

**图3:**
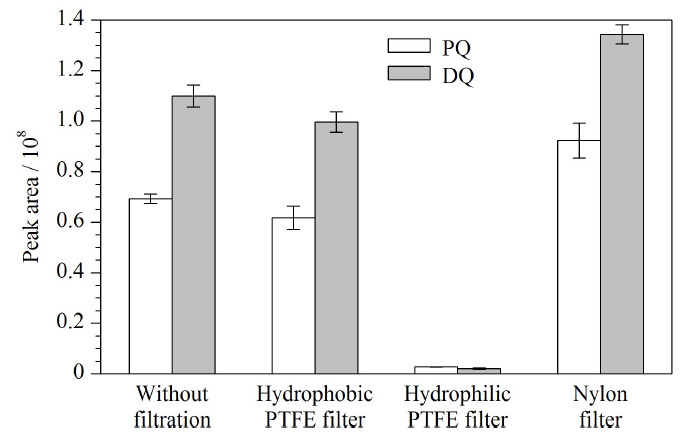
不同类型滤膜对PQ和DQ峰面积的影响(*n*=3)

#### 2.3.2 定容液的选择

在HILIC色谱柱分析待测化合物时,溶剂效应通常会比较明显。溶剂效应常常体现在色谱保留时间、色谱峰形和峰面积等方面。在本研究中,分别考察了不同比例乙腈-水溶液对PQ和DQ分析过程的影响。由实验结果可知,当定容液为纯水时,PQ的色谱峰有明显的前伸现象,由此可知采用纯水作为定容液分析PQ不合适。当乙腈-水(1∶5, v/v)作为定容液时,前伸现象有了显著改善,但相比乙腈-水(1∶1, v/v)以及更高比例乙腈-水作为定容液时,PQ的色谱峰对称性有所欠缺。这一现象在DQ的检测过程中也极其相似。可能的原因是,HILIC模式下目标分析物先被分散至色谱柱固定相周围的吸附水层,然后通过极性相互作用或静电相互作用进行吸附,但样品溶液中水的比例过高会影响目标分析物至吸附水层的分散过程及后续的吸附过程,从而影响色谱峰形。

更为重要的是,定容液组成对PQ和DQ的峰面积影响更为明显。当纯水作为定容液时,PQ和DQ的峰面积较小;当乙腈-水(1∶5, v/v)作为定容液时,PQ和DQ的峰面积有了明显增加;当乙腈-水(1∶1, v/v)作为定容液时,PQ和DQ的峰面积达到最大,进一步增加定容液中乙腈的比例,PQ和DQ的峰面积基本保持不变。考虑到PQ和DQ的水溶性好,定容液中加入适量的水有利于氮吹过后PQ和DQ的溶解,因此,最终选择乙腈-水(1∶1, v/v)作为定容液。

#### 2.3.3 进样瓶材质的影响

实验发现,进样瓶可能对溶液中PQ和DQ有吸附作用,从而影响检测结果。本研究分别选择了普通玻璃进样瓶和聚丙烯进样瓶,将PQ和DQ的混合标准溶液装于两种材质进样瓶中,考察峰面积随时间的变化,结果见图4。由图4可知,对于玻璃材质进样瓶,经6 ℃条件下放置16 h, PQ和DQ分别损失了85.4%和91.0%;放置38 h,两者分别损失了93.2%和94.7%;继续放置60 h, PQ和DQ未见进一步损失。对于聚丙烯材质进样瓶,放置60 h时间内PQ和DQ均未见显著变化。可能的原因是,玻璃材质进样瓶表面存在大量Si-OH,很容易与溶液中带正电荷的PQ和DQ发生静电引力作用,从而导致样品溶液中PQ和DQ被玻璃进样瓶壁吸附而损失。因此,后续实验均采用聚丙烯材质进样瓶。

**图4:**
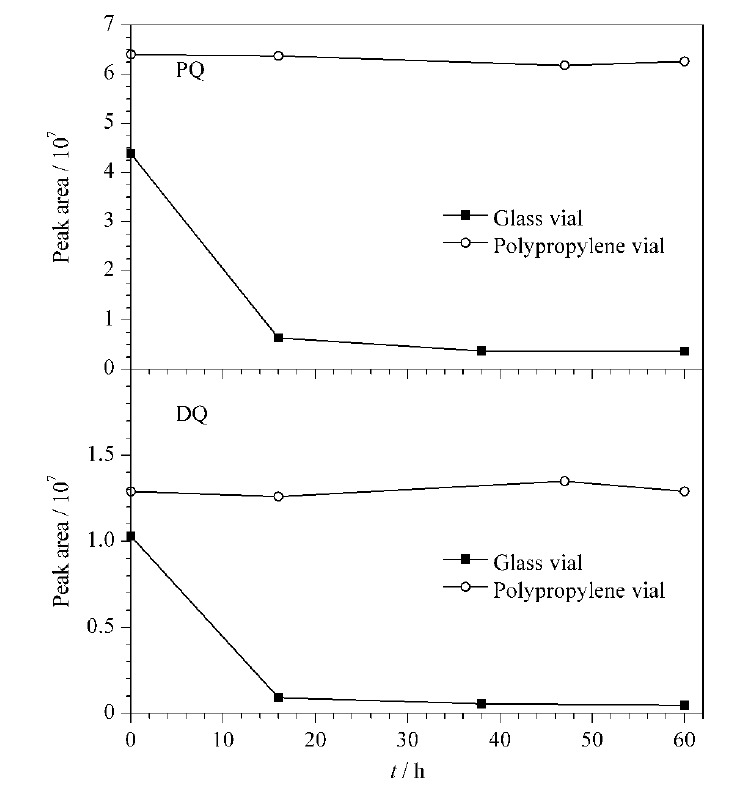
进样瓶材质对目标分析物稳定性的影响

#### 2.3.4 溶液pH值的影响

由于PQ和DQ表面带正电荷,按照异种电荷相互吸引的原理,可选择MCX或WCX固相萃取柱作为净化柱。实验结果表明,MCX固相萃取柱在净化尿液中PQ和DQ时,其加标回收率<30%,大部分PQ和DQ难以从MCX小柱上洗脱下来。可能的原因是MCX小柱的填料表面由苯磺酸根修饰,其对带正电的PQ和DQ的吸附能力很强。而WCX小柱的吸附填料为羧基功能化高分子材料,其对PQ和DQ的吸附能力相对较弱,相比MCX小柱更容易被洗脱下来。

然而,在实验中发现尿液样品的pH值对净化性能有显著影响。本研究对比了3个pH缓冲体系下(分别是pH=4.00邻苯二甲酸氢钾缓冲液、pH=6.86混合磷酸盐缓冲液和pH=9.18硼酸缓冲液)WCX小柱对尿液加标样品中PQ和DQ净化性能的影响,实验结果见图5。由图5可知,当溶液pH=4.00时,PQ和DQ的峰面积较小;而随着溶液pH增加,两者的峰面积增大,且溶液pH=6.86和9.18时峰面积达到平台值。

**图5:**
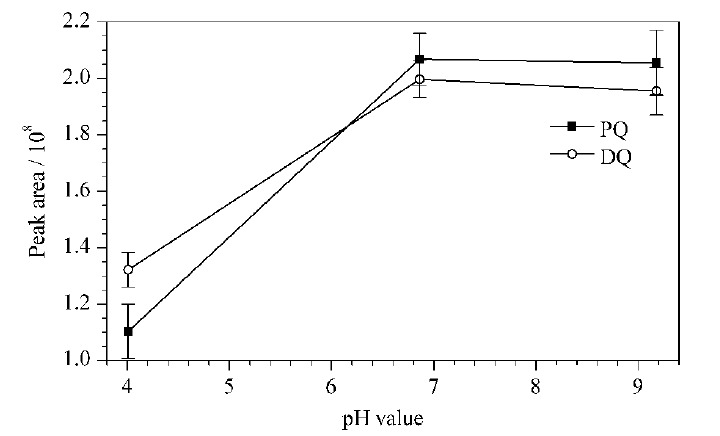
样品溶液pH值对PQ和DQ净化性能的影响(*n*=3)

可能的原因是,样品溶液的pH值能改变WCX小柱吸附材料表面羧基官能团和待测化合物官能团的解离情况,从而改变其对溶液中PQ和DQ的吸附行为。然而,PQ和DQ在不同pH条件下均为季铵阳离子,因此只需研究不同pH值下WCX吸附材料表面羧基(-COOH)的形态变化。WCX材料表面羧基的p*K*_a_约为4.20。根据Henderson-Hasselbach方程式^[23]^,对于弱酸R-COOH,当溶液pH<p*K*_a_时,WCX材料表面只有少量离子化的羧基(<50%)可以与带正电的PQ和DQ发生静电引力作用,此时两者的峰面积较小;当溶液pH≥6.20(大于p*K*_a_值2个单位以上)时,WCX材料表面99%以上的羧基呈电离状态,可与PQ和DQ之间发生较强的静电引力作用。上述实验中混合磷酸缓冲液和硼酸缓冲液的pH值均大于羧基p*K*_a_值2个单位,所以两者的实验结果中PQ和DQ的峰面积最大。

同理,在进行PQ和DQ洗脱时,需要调节溶液pH≤2.20(即小于p*K*_a_值2个单位以上),此时WCX材料表面99%羧基呈电中性状态,静电引力作用消失,PQ和DQ能顺利地从材料表面洗脱下来。因此,本研究选择甲酸-乙腈(2∶98, v/v)作为洗脱剂。

### 2.4 方法验证与确认

#### 2.4.1 基质效应评价

分别采用空白尿液的前处理液和乙腈-水(1∶1, v/v)配制PQ和DQ质量浓度为1.0~200.0 μg/L的基质匹配标准溶液和溶剂标准溶液,测定PQ和DQ的峰面积,得到基质匹配工作曲线和溶剂工作曲线。根据公式基质效应(*η*值)=(基质匹配标准曲线斜率*k*_a_-溶剂标准曲线斜率*k*_b_)/溶剂标准曲线斜率*k*_b_×100%^[24⇓-26]^计算,得到PQ和DQ的*η*值分别为100%和50%。由此可知,PQ和DQ分别为强基质效应和中等强度基质效应,需要采用基质匹配工作曲线进行定量分析才能得到准确的检测结果。

#### 2.4.2 线性方程、检出限和定量限

采用空白尿液的前处理液配制PQ和DQ质量浓度为1.0、5.0、10.0、50.0、100.0和200.0 μg/L的基质匹配标准溶液,测定PQ和DQ峰面积。以峰面积(*Y*)对化合物的质量浓度(*X*, μg/L)进行线性回归。结果表明,PQ和DQ在1.0~200.0 μg/L范围内线性关系良好,回归方程分别为*Y*=2.0×10^6^*X*-2.0×10^5^和*Y*=3.0×10^6^*X*+3.0×10^5^,相关系数均大于0.998。

在空白尿液样品中分别加入系列低浓度的PQ和DQ,采用1.2.1节步骤进行样品前处理,UPLC-HRMS测定得到信噪比(*S/N*)≥3和*S/N*≥10时的样品浓度作为检出限(LOD)和定量限(LOQ)。结果表明PQ和DQ的LOD均为0.2 μg/L, LOQ均为0.6 μg/L。

#### 2.4.3 加标回收率和精密度

分别移取1.0 mL空白尿液样品,加入适量PQ和DQ的混合标准溶液,使样品中两者的加标浓度分别为1.0、20.0、100.0和200 μg/L,如1.2.1节步骤进行样品前处理,然后采用UPLC-HRMS检测,每个水平平行测定6次。由表2可知,PQ和DQ的平均加标回收率分别为85.8%~101%和80.3%~86.9%,精密度(RSD)分别为0.8%~5.1%和0.9%~4.2%,能满足实验室日常中毒检测的要求。

**表2:** 尿液中PQ和DQ的加标回收率和精密度(*n*=6)

Analyte	Added/(μg/L)	Found/(μg/L)	Recovery/%	RSD/%
PQ	1.0	1.01	101	5.1
	20.0	18.4	92.2	2.8
	100.0	92.3	92.3	0.8
	200.0	171.6	85.8	1.3
DQ	1.0	0.861	86.1	4.2
	20.0	16.9	84.5	2.6
	100.0	80.3	80.3	0.9
	200.0	173.8	86.9	2.8

### 2.5 中毒样品分析

应用本研究建立的方法对临床中毒尿液样品进行检测,其中有1例中毒病例的尿液样品经UPLC-HRMS确证为DQ中毒。本研究跟踪监测了该病例治疗过程中尿液样品中DQ的浓度变化。结果显示,血液灌流前尿液中DQ含量非常高,为100 mg/L;经1次血液灌流后,尿液中DQ含量显著降低至4.79 mg/L;经5次血液灌流治疗后,尿液中DQ含量降低至0.17 mg/L,但停止血液灌流24 h后,尿液中DQ含量有所回升,为1.30 mg/L。因此,本研究建立的基于WCX固相萃取-UPLC-HRMS的分析方法可用于临床中毒病例的确证和治疗过程中浓度的跟踪监测,为临床精准治疗提供技术支撑。

## 3 结论

本文系统考察了尿液中PQ和DQ检测过程中的各种影响因素,如滤膜的选择、进样瓶材质、定容液组成、上样液pH值和基质效应等,建立了基于弱阳离子交换固相萃取净化-超高效液相色谱-高分辨质谱测定尿液样品中PQ和DQ残留的分析方法,并将本文建立的方法应用于中毒样品的确证与临床治疗过程中的浓度监测。该方法快速、简便、准确和灵敏,适用于临床中毒样品的确证分析与浓度检测。
